# Immune Protection of Rhoptry Protein 21 (ROP21) of *Toxoplasma gondii* as a DNA Vaccine Against Toxoplasmosis

**DOI:** 10.3389/fmicb.2018.00909

**Published:** 2018-05-08

**Authors:** Zhenchao Zhang, Yuhua Li, Mingyong Wang, Qing Xie, Pengju Li, Suqiong Zuo, Lingmin Kong, Chenxing Wang, Shuai Wang

**Affiliations:** ^1^School of Basic Medical Sciences, Xinxiang Medical University, Xinxiang, China; ^2^The First Affiliated Hospital of Xinxiang Medical University, Xinxiang, China; ^3^Henan Key Laboratory of Immunology and Targeted Therapy, Henan Collaborative Innovation Center of Molecular Diagnosis and Laboratory Medicine, School of Laboratory Medicine, Xinxiang Medical University, Xinxiang, China

**Keywords:** *T. gondii*, rhoptry proteins 21, DNA vaccine, animal challenge, protective immunity

## Abstract

*Toxoplasma gondii* rhoptry proteins (TgROPs) are the major targets as key molecules for immunodiagnosis as well as immunoprophylaxis because of their initial presentation to the host immune system. In this work, it was aimed at evaluating the protection effect of TgROP21 DNA vaccine on experimental mice subjected to *T. gondii* challenge. The gene sequence encoding TgROP21 was inserted into the eukaryotic expression vector pVAX I, and western blotting indicates that the lysate of BHK cells transfected with pVAX-TgROP21 was specifically recognized as a band of about 82.6 kDa by serum obtained from a *T. gondii* infected chicken. The efficacy of intramuscular vaccination of BALB/c mice three times at weeks 0, 2, and 4 with pVAX-ROP21 was analyzed. The levels of IgG, IgG1, and IgG2a among pVAX-ROP21 vaccinated animals were integrally increased. It was uncovered by cytokine profile analyses that IFN-γ was significantly increased, while no significant changes were detected in interleukin-2 (IL-2), interleukin-4 (IL-4), and interleukin-10 (IL-10). Additionally, we found that immunization with pVAX-ROP21 significantly prolonged survival time (13.50 ± 1.65 days) after challenge infection with the virulent *T. gondii* RH strain, in comparison to those of control animals (died within 10 days). Moreover, the number of brain cysts (1475 ± 163) in the animals subjected to pVAX-TgROP21 vaccination decreased remarkably (*P* < 0.05) compared to the blank control mice (2333 ± 473), and the size of brain cysts in pVAX-TgROP21 group was significantly smaller than the groups of blank, PBS and pVAXI. It was indicated that intense cell-mediated and humoral immunity was triggered and defense against *T. gondii* was partially induced after vaccination by TgROP21.

## Introduction

*Toxoplasma gondii*, which belongs to phylum Apicomplexa, lives an obligatory endotrophic life cycle and infects multitudinous host animals which include almost all endotherm organisms. As a kind of parasitosis which is spread by animals and distributed all around the world, toxoplasmosis threatens the health of human and livestock seriously (Calderaro et al., 2006). About 30% worldwide population have been infected by *T. gondii* (Montoya and Liesenfeld, 2004). The toxicity intensity is used to evaluate the virulence and classify various strains of *T. gondii*. The host will suffer from acute toxoplasmosis or even die in case of being infected by virulent strains, while the symptoms are probably unapparent with generation of cysts in some hosts in case of weak strains (Elmore et al., 2010; Dubey et al., 2012). In addition, toxoplasma infections result in abortion, stillbirth, as well as death of neonates which leads to great financial loss (Edwards and Dubey, 2013; Chessa et al., 2014). The main spreading route to human is infected domestic animals (Gebremedhin et al., 2013).

Although some chemical drugs (atovaquone, sulfadiazine, and pyrimethamine) could control *T. gondii* acute infections, they could not eliminate the chronic infection (Innes, 2010). Additionally, *T. gondii* with resistance to drugs has appeared and drug residue in animal-derived foods has occurred more and more extensively (Nagamune et al., 2007; Garrison and Arrizabalaga, 2009; Kur et al., 2009). Consequently, new and effective vaccines are urgently needed to fight against *T. gondii*. Thus, it is imperative to develop effective and safe vaccines against *T. gondii* infections in humans and animals. Stock-raising industry will also benefit from this measure through reducing financial loss (Kur et al., 2009).

A variety of contagious diseases which include hepatitis B are successfully prevented by vaccines (Zhang et al., 2013). By now, inactivated vaccine, subunit vaccine, as well as cocktail vaccine with multiple antigens have been developed to prevent infection of *T. gondii* (Kur et al., 2009). Toxovax^®^, the only available commercial vaccine, is based on live-attenuated *T. gondii* S48 strain and is licensed only for use in sheep to prevent abortion (Buxton and Innes, 1995). However, it is limited to be further explored in food-producing animals or humans in view of the safety concerns on its reverting to a virulence wild type. Consequently, DNA vaccines against *T. gondii* have gradually become a hotspot in the area of preventing and treating toxoplasmosis with higher priority over subunit vaccines, inactivated vaccines, as well as drugs. For the past few years, a great deal of antigen candidates has been screened out for *T. gondii* DNA vaccines (Laddy and Weiner, 2006; Saha et al., 2011; Zhang et al., 2013).

It was revealed by analyses of comparative genomics that the diversification and amplification of rhoptry organelle proteins (ROPs), which act as a determinant of secretory pathogenesis, exert an essential role in distinguishing genomic information of various coccidian pathogens (Lorenzi et al., 2016). ROP molecules play important roles in invasion, in establishing the replicative niche of the parasitophorous vacuole, in host cell manipulation, and in resisting host IFN-γ-stimulated innate immunity (Håkansson et al., 2001; Bradley et al., 2005; Boothroyd and Dubremetz, 2008). Some studies showed that 19 ROPs exhibited activity of protein kinases, such as ROP38, ROP21, ROP18, and ROP16, while the ROP16 and ROP18 were confirmed as rod-like secretory protein kinases (Butcher et al., 2011). Moreover, these ROPs catalyze phosphorylation on themselves as well as their down-stream protein targets and the latter exert determinate role in *T. gondii* infecting hosts (Yamamoto et al., 2009; Ong et al., 2010). In some studies, aligning results revealed partial sequence similarity in amino acids between ROP54 and ROP18 as well as ROP16, indicating analogous function exerted by ROP16, ROP18, and ROP54 in host cells.

In addition, genes which encode specific ROPs of *T. gondii* have been regarded as ideal materials for producing anti-*T. gondii* DNA vaccines. Some studies suggested ROP1 as one of potent stimulators of mouse cell-mediated and humoral immunities (Ferra et al., 2015). It was revealed by the work of Yuan et al. (2011a; 2011b) that ROP16 would be used to produce anti-*T. gondii* DNA vaccines and cytotoxic T cell (CTL) specific to *T. gondii* was enhanced by the plasmid built by ROP18 gene.

However, few functions of *T. gondii* rhoptry protein 21 (TgROP21) have been reported in previous studies. The aim of this study was evaluating mouse immunoreaction after injecting an innovative DNA vaccine constructed by TgROP21 and eukaryotic expressing vector, as well as evaluating the capacity of TgROP21 acting as a candidate of anti-*T. gondii* DNA vaccines which prevented *T. gondii* infection among BALB/c mice.

## Materials and Methods

### Ethics Statements

The guidance of the Animal Ethics Committee, Xinxiang Medical University, Henan, China (Reference No. 2015016), was followed all through the experiments of this study. The pain of experimental animals was minimized by all efforts. In order to reduce the pain or distress of experimental mice, humane endpoints were used by euthanasia. Normally, the mice were placed in an environment over the exposure time of 5 min with the concentration of 60–70% CO_2_. And after that, sometimes, the method of cervical dislocation was applied to ensure effective euthanasia.

### Sequence Analysis

In order to assess the possibility of TgROP21 as a candidate vaccine antigen, DNASTAR software (Madison, WI, United States) was used to predict the surface probability, antigenic index, hydrophilic plot, and flexible region.

### Mice and Cell Culture

Five-week-old female BALB/c mice (*n* = 160) were purchased from Beijing Vital River Laboratory Animal Technology Co., Ltd. (Beijing, China) and maintained under specific-pathogen-free standard conditions.

Baby hamster kidney (BHK) cells or human foreskin fibroblast (HFF) cells were grown and maintained in Dulbecco’s Modified Eagle’s Medium (DMEM; Gibco, Beijing, China) supplemented with L-glutamine, 10% (BHK) or 2% (HFF) dialyzed fetal bovine serum (FBS; Gibco, Beijing, China), 100 IU/ml penicillin, and 100 μg/ml streptomycin in a humidified chamber containing 5% CO_2_ at 37°C.

### Parasites and Preparation of Soluble Tachyzoite Antigens (STAg)

*Toxoplasma gondii* RH strain (Type I) and PRU strain (Type II) were provided by the Department of Human Parasitology, Xinxiang Medical University, Henan, China. The tachyzoites of Type I was gathered from HFF cells. The PRU strain of *T. gondii* was maintained by passage of cysts in BALB/c mice. According to the requirements of biological safety, the recovery, cultivation, and infection of *T. gondii* were operated by professional personnel (Wang et al., 2014).

Soluble tachyzoite antigen was prepared from *T. gondii* tachyzoites as described previously (Zhang et al., 2016). Briefly, tachyzoite cells in lyses buffer with 1 mM phenylmethanesulfonyl fluoride (PMSF) were frozen under the temperature of -20°C and thawed under 4°C for three times with the aim of disruption after purification. Subsequently, the obtained lysate was subjected to ultrasonic dispersion under the speed of 60 W/s on ice and separated by centrifuge at 14,000 ×*g* for half an hour under the temperature of 4°C. The obtained supernatant samples were combined and subjected to filtration sterilization and the final STAg was preserved by small portions under the temperature of -70°C before analyses. Bradford method was used to determine the concentration of protein sample with the standard of bovine serum albumin (BSA).

### Constructing the Plasmid for DNA Vaccine

RT-PCR was conducted to amplify the entire open reading frame (ORF) of TgROP21 (GenBank accession no. XM_018781319.1). The forward primer sequence was 5′-*CGGGGTACC*ATGGTTCACGGGCAAATGC-3′ and the reverse primer sequence was 5′-*CCGGAATTC*CTAGTCTTCTTCTGTCGAGCCGAT-3′ and the inclined letters in these two sequences were used to indicate the respective restriction site of *Kpn* I and *ECO*R II. TgROP21 fragments amplified by RT-PCR were initially ligated to pMD19-T vector (TaKaRa, Dalian, China) for cloning. Subsequently, the sequence was digested from pMD-TgROP21 by *Kpn* I and *ECO*R II and ligated to the same site of pVAXI vector (Invitrogen, Carlsbad, CA, United States) to construct the recombinant plasmid of pVAX-TgROP21 for sub-cloning. pVAX-TgROP21 was transfected into DH5α cells of *Escherichia coli* and the existence of which was proven by DNA sequencing and restriction endonuclease experiment. A commercial kit (TianGen, Beijing, China) was used to isolate pVAX-TgROP21 and eliminate endotoxin and OD260/280 measured by a spectrophotometer was used to determine the concentration of product.

### *In Vitro* Expressions of Recombined Plasmids

Above all, BHK cells were transferred into a six-well plate (Costar, NY, United States) and transfected by 5 μg of pVAX-TgROP21 mixed with Lipofectamine 3000 regent (Invitrogen, Carlsbad, CA, United States) under the confluency of 80–90% based on the manufacturer’s guidelines. Meanwhile, 5 μg of empty vector pVAXI was used to transfect the cells in negative control group. In brief, empty vectors and recombined plasmids were separately added into DMEM which contained 10 μg/ml Lipofectamine 3000 without antibiotic drugs or FBS, and incubated under ambient condition for half an hour. Subsequently, BHK cells were treated by mixed plasmids and lipofectamine for 6 h under the condition of 37°C and 5% CO_2_. After that, DMEM containing mixture was replaced for new culture medium and the cells were incubated under the previous condition for another 48 h. Subsequently, RIPA Lyses Buffer (50 mM Tris pH 7.4, 150 mM NaCl, 0.1% SDS, 1% sodium deoxycholate, and 1% Triton X-100) which contained 1 mM PMSF as protease inhibitor was utilized to treat the cells on ice and the lysates were separated by centrifuge under the speed of 13,000 ×*g* for 10 min.

On the basis of modification by Schagger (2006), it was preferable to isolate lysate protein by electrophoresis using 10% of Tricine-SDS-PAGE (Tricine-sodium dodecyl sulfate-polyacrylamide gel electrophoresis). After that, the gel was transferred onto polyvinylidene difluoride (PVDF) membrane and western blot was utilized to detect gene expressions with the first antibody of chicken anti-*T. gondii* polyclonal antibody and the second antibody of goat anti-chicken IgG antibody labeled by HRP (SouthernBiotech, Birmingham, AL, United States). Subsequently, the PVDF membrane carrying protein spots was immersed into DAB Reagent (Boshide Biotech Co, Wuhan, China) until further detection.

### Inoculating and Challenging BALB/c Mice Against *T. gondii*

With the aim of evaluating the immunogenic property of recombined plasmid, 160 BALB/c mice were separated into four groups at random. Initially, the plasmid was subjected to dilution and suspension using sterilized PBS till the terminal concentration of 1 μg/μl. All animals in three test groups were subjected to thrice intramuscular injection containing 100 μg of recombined plasmids, empty vectors, or equal volume of PBS separately at zeroth, second, and fourth week. The animals in negative control group did not receive any inoculation. Five animals from each of the four groups were subjected to blood sampling from tail veins on the zeroth, second, fourth, and sixth week, respectively. The animals were vaccinated on the following day and subjected to blood sampling again 14 days later. Serum samples were separated from whole blood and preserved under the temperature of -20°C in order to evaluate levels of antibodies and cytokines. The rest 20 animals of each test and control groups were subjected to challenging 14 days after final inoculation, in which 10 were subjected to intraperitoneal challenging using 1 × 10^3^ tachyzoite cells of RH *T. gondii*, while the other 10 animals were challenged intraperitoneally (i.p) using 10 cysts of *T. gondii* PRU strain. The animals subjected to challenging by RH *T. gondii* were under observation and the record of surviving duration was kept. The mice were sacrificed immediately by using the gas of CO_2_, when they showed signs of illness.

After 1 month, the survival rate of mice challenged with the PRU strain was observed and the brains of these animals were removed and grinded by 1 ml of PBS; 10 μl brain mixture from each animal was used to count cyst quantity for thrice. The size of cysts in each group observed with a microscope. The size of brain cysts (*n* = 10) was presented by the diameter of cysts. The diameter of cysts was measured by the camera control software (ToupeView) and the size of the diameter of cysts was presented by pixel (px). Cysts’ decrease ratio was calculated as follows: (the number of cysts from the blank control mice – vaccinated mice)/the blank control mice × 100%, and the decrease ratio of size of cysts was calculated as follows: (the size of cysts from the blank control mice – vaccinated mice)/the blank control mice × 100%.

### Determining Antibody Levels Through ELISA

According to previous literature, indirect ELISA (enzyme-linked immunosorbent assays) was utilized to determine mouse serum antibody levels (Hassan et al., 2014a). Briefly, 100 μl 50 mM carbonate buffer which contained 10 μg/ml STAg with the pH value of 9.6 was added into each well of a microtiter plate (Costar, New York, NY, United States) for coating all through the night under the temperature of 4°C. After washing the plates thrice, 3% of BSA was utilized to block them for 2 h under the temperature of 37°C, and then PBS diluents of serum samples (ratio = 1/10) were used to incubate them for 2 h under the same temperature. After that, the second antibody including goat anti-mouse IgG, IgG1, and IgG2a conjugated with HRP (SouthernBiotech, Birmingham, AL, United States) were added into the plates for incubation. In the end, 3,3,5,5-tetramethylbenzidine was added into and then incubated with the plates for 20 min before being terminated by the addition of 2 M sulfuric acid. An automatic ELISA reader (MULTISKAN FC, Thermo scientific, Waltham, MA, United States) was utilized to measure the absorbing values at 450 nm and all tests were conducted for thrice.

### Determination of Cytokines

According to the above-mentioned procedures, the serum samples of all subjects were prepared to determine the expression of cytokines. Interleukin-2/4/10 (IL-2, IL-4, IL-10) as well as IFN-γ were measured using ready ELISA kits according to the manufacturer’s instructions (Boster Systems, Wuhan, China). Cytokine concentrations were determined by reference to standard curves constructed with known amounts of mouse recombinant IL-2, IL-4, IL-10, and IFN-γ. The analysis was performed with the data from three independent experiments.

### Statistics Analyses

IBM SPSS 20.0 Data Editor (SPSS Inc., Chicago, IL, United States) was utilized to perform entire statistics analyses. One-way ANOVA was used to evaluate data varieties between groups such as cytokine expressions and responding levels of antibodies. Kaplan–Meier method was applied to the comparison of surviving periods. *P* value < 0.05 was considered as statistically significant.

## Results

### Epitope Analysis

**FIGURE 1:**
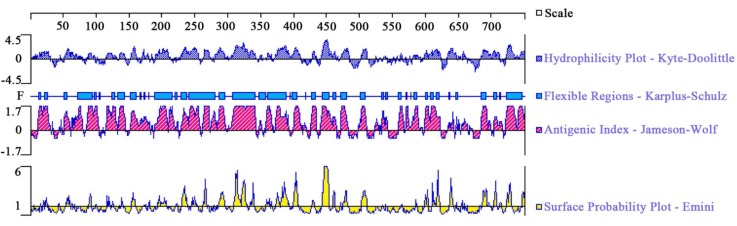
The linear-B cell epitopes of TgROP21 predicted by DNASTAR in hydrophilicity plot, flexible regions, antigenic index, and surface probability rules.

DNASTAR was used to analyze the protein of TgROP21 of surface probability, antigenic index, hydrophilic plot, as well as flexible region. According to **Figure 1**, most regions of TgROP21 protein were hydrophilicity plots and flexible regions, and TgROP21 exhibited ideal surface probability and antigenic index which indicated a promising prospect of producing vaccines with it.

### Identification of the Eukaryotic Expression Plasmid

**FIGURE 2:**
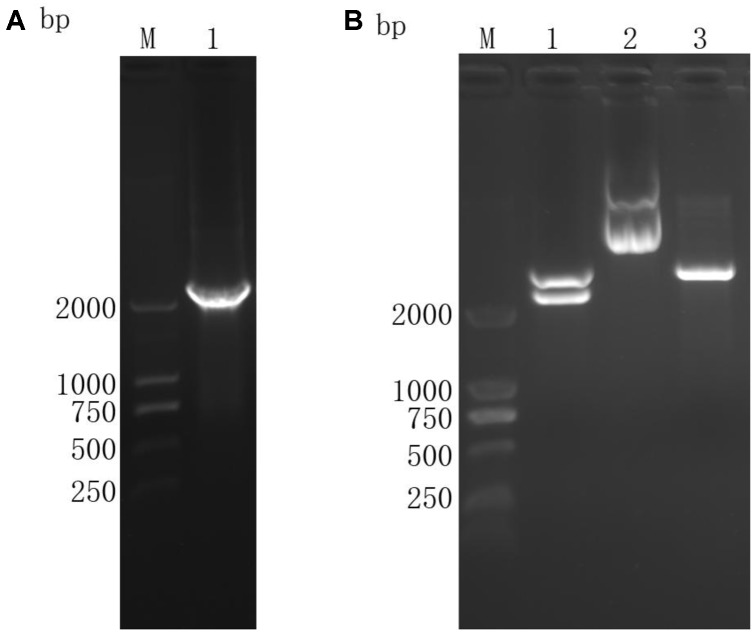
Agarose gel electrophoresis of TgROP21 ORF and identification of recombinant plasmid pVAX-TgROP21 digested by *Kpn*I and *ECO*RI. **(A)** (Lane M) DNA molecular weight marker DL 2000 (ordinate values in bp); (Lane 1) the ORF of TgROP21. **(B)** (Lane M) DNA molecular weight marker DL 2000 (ordinate values in bp); (Lane 1) the recombinant plasmid pVAX-TgROP21 digested by *Kpn*I and *ECO*RI; (Lane 2) the recombinant plasmid pVAX-TgROP21; (Lane 3) the plasmid of pVAXI vector digested by *Kpn*I and *ECO*RI.

The ORF was obtained by RT-PCR (**Figure 2A**) and the target fragment sized as 2251 bp (**Figure 2B**) was confirmed through restriction enzyme digestion. The fragments cut off from plasmids were sequenced and confirmed as TgROP21 ORF, indicating successful construction of the gene vaccine pVAX-TgROP21.

### Using Western Blot to Identify Product Expression

**FIGURE 3:**
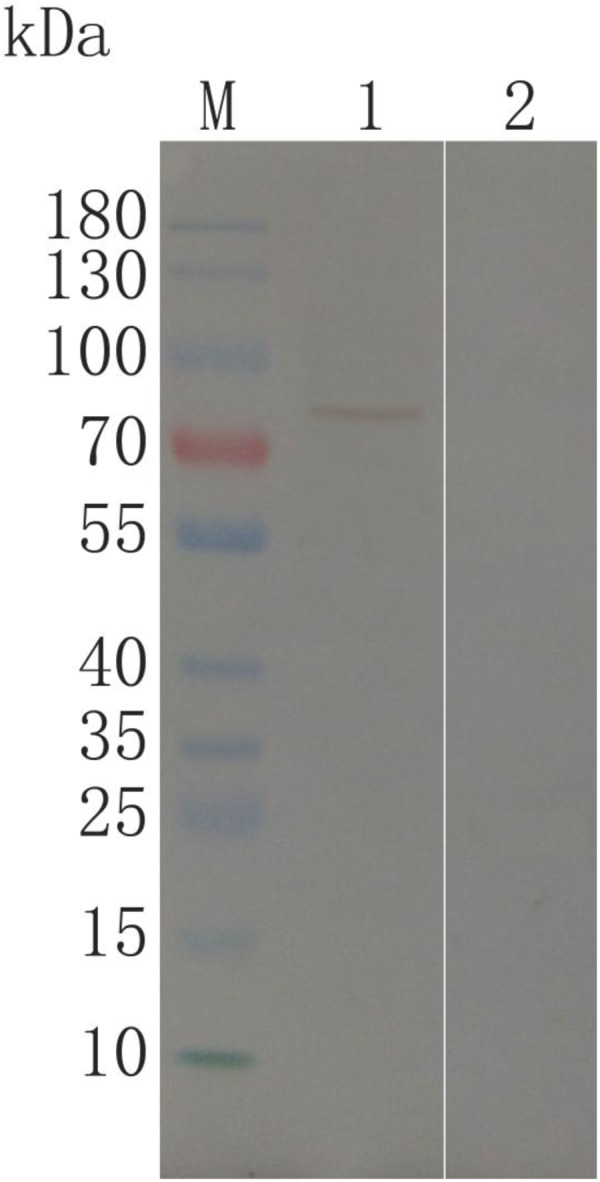
Immunoblot for identification of TgROP21 in BHK cells. (Lane M) protein Mark (ordinate values in kDa); (Lane 1) lysates of BHK cells transfected with pVAX-TgROP21 were probed with chicken anti-*T. gondii* sera; (Lane 2) lysates of BHK cells transfected with the plasmid of pVAXI vector probed with chicken anti-*T. gondii* sera.

The BHK cells subjected to pVAX-TgROP21 transfection were lyzed and separated by western blot (**Figure 3**). The specific protein resulting from pVAX-TgROP21 transfection was identified by chicken-derived anti-*T. gondii* serum as a band of about 93 kDa, which was consistent with the deduced size. On the contrary, this protein was not found in cells subjected to pVAX I transfection.

### Humoral Immunoreactions

**FIGURE 4:**
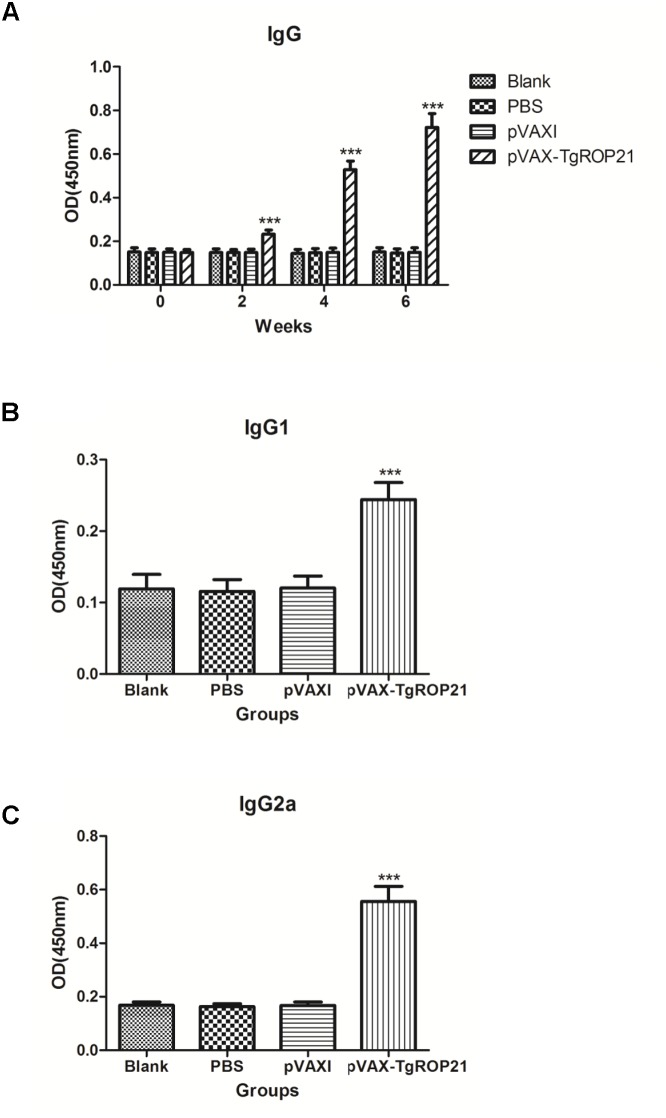
The dynamics of humoral response in BALB/c mice induced by DNA vaccination pVAX-TgROP21. The BALB/c mice were randomly divided into four groups of five mice each (*n* = 5). The BALB/c mice in group were immunized with pVAX-TgROP21, pVAX I, and PBS and the rest of the group as blank controls. The titers of IgG were detected on weeks 0, 2, 4, and 6, and the IgG subclass IgG1 and IgG2a were detected 2 weeks after the last immunization. The results were expressed as mean ± *SD* with respect to absorbance at 450 nm. Statistically significant differences (*P* < 0.05), (*P* < 0.01), and (*P* < 0.001) were indicated by (^∗^), (^∗∗^), and (^∗∗∗^) in different groups at the same time point, respectively. **(A)** IgG. **(B)** IgG1. **(C)** IgG2a.

With the aim of evaluating variation of antibody levels resulting from three sequential inoculation of DNA vaccine, the total quantities of IgG after each injection and the distributions of IgG1 and IgG2a isotypes 14 days after the final injection were tested. The serous IgG levels of animals subjected to pVAX-TgROP21 injection were higher with statistical significance than those of controls (*P* < 0.001). Moreover, the OD value of IgG was increasing along with the continuous injection. None of the control groups exhibited different IgG level from the other two with statistical significance (**Figure 4A**).

Compared to control groups, animals injected with pVAX-TgROP21 exhibited highest IgG1 and IgG2a expressions (*P* < 0.001; **Figures 4B,C**), in which IgG2a was apparently predominant to IgG1, indicating that pVAX-TgROP21 vaccine induced Th1-type cell immunoreaction.

### Expressions of Cytokines

**FIGURE 5:**
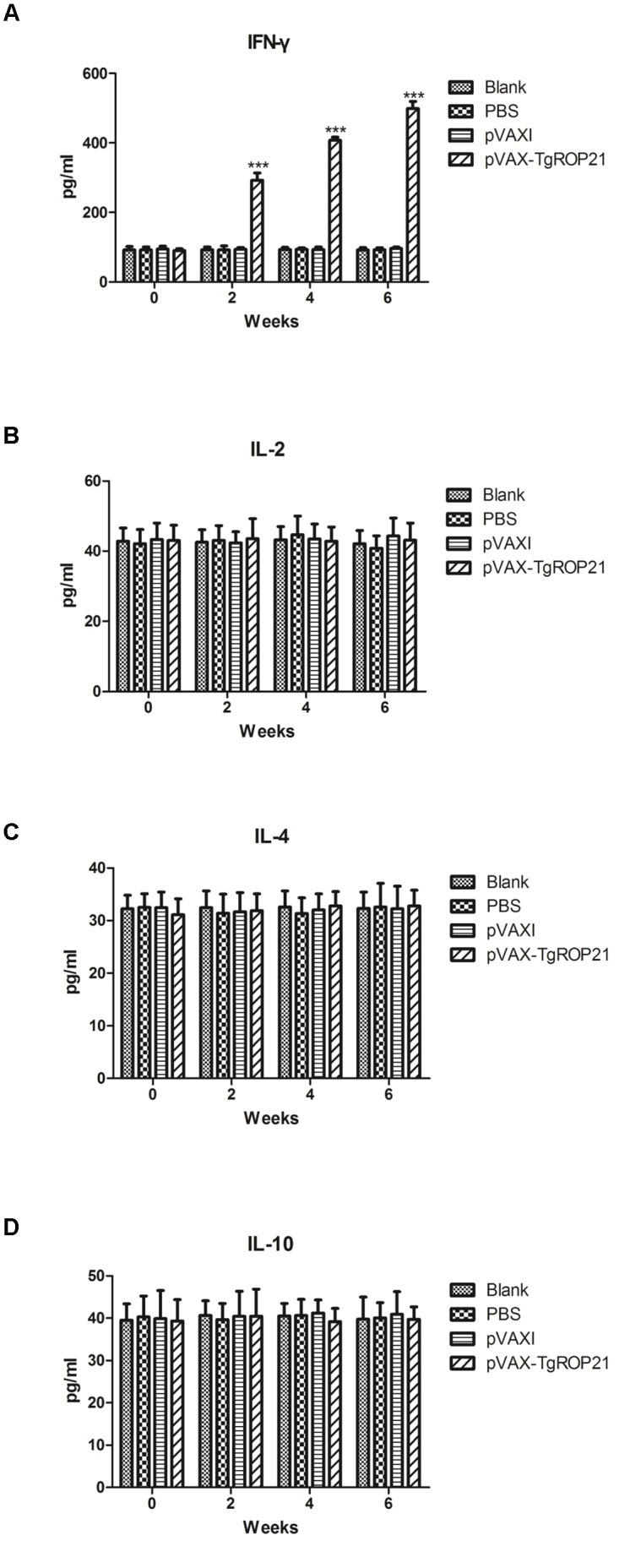
Cytokine production. The BALB/c mice were randomly divided into four groups of five mice each (*n* = 5). The BALB/c mice in group were immunized with pVAX-TgROP21, pVAX I, and PBS and the rest of the group as blank controls. The production levels of cytokine were determined by antigen-captured ELISA on weeks 0, 2, 4, and 6 and the comparison results were expressed as mean ±*SD* of pg/ml. Statistically significant differences (*P* < 0.05), (*P* < 0.01), and (*P* < 0.001) were indicated by (^∗^), (^∗∗^), and (^∗∗∗^) in different groups at the same time point, respectively. **(A)** IFN-γ. **(B)** IL-2. **(C)** IL-4. **(D)** IL-10.

The quantities of IFN-γ and IL-2/4/10 in each test group were measured using serum specimens gathered on the zeroth, second, fourth, and sixth week. It was revealed by **Figure 5A** that IFN-γ quantities among animals subjected to pVAX-TgROP21 injection were remarkably increased in comparison to those of controls on the second, fourth, and sixth week after inoculation (*P* < 0.001). However, there were no significant differences of the IL-2, IL-4, and IL-10 between the immunized and control groups (**Figures 5B–D**).

### Evaluation on the Protection of DNA Vaccination on Inoculated Animals Against Pathogen Challenge

**FIGURE 6:**
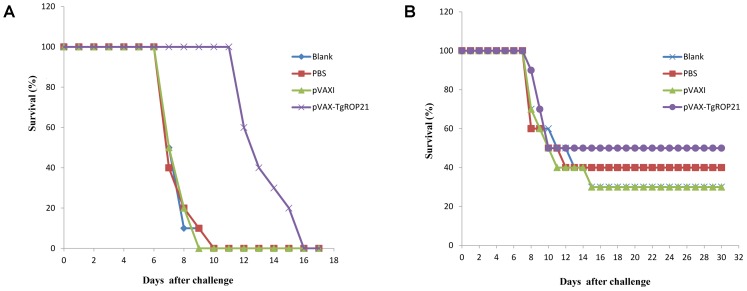
Survival curve of mice after challenge infection with *T. gondii* RH strain and PRU strain. Mice were, respectively, challenged with **(A)** 10^3^ tachyozoites of the RH strain intraperitoneally and **(B)** 10 cysts of *T. gondii* PRU strain intragastrically 2 weeks after the third immunization.

With the aim of evaluating the protection of DNA vaccination on immunized animals against pathogen, the surviving duration and cyst number were recorded after the animals were infected by 10^3^ tachyzoite cells of RH *T. gondii* and 10 cysts of PRU strain. Survival curves of different groups of mice were shown in **Figure 6**. The surviving duration of animals injected with pVAX-TgROP21 (13.50 ± 1.65 days, *P* < 0.05) was remarkably longer than that of subjects receiving PBS or empty vector (**Figure 6A**). As the result in **Figure 6B**, five mice remained survived in the group of pVAX-TgROP21, 4 weeks after oral challenge with 10 cysts of the *T. gondii* PRU strain. However, the remaining mice in the group of blank, PBS, and pVAXI were 3, 4, and 3, respectively.

**FIGURE 7:**
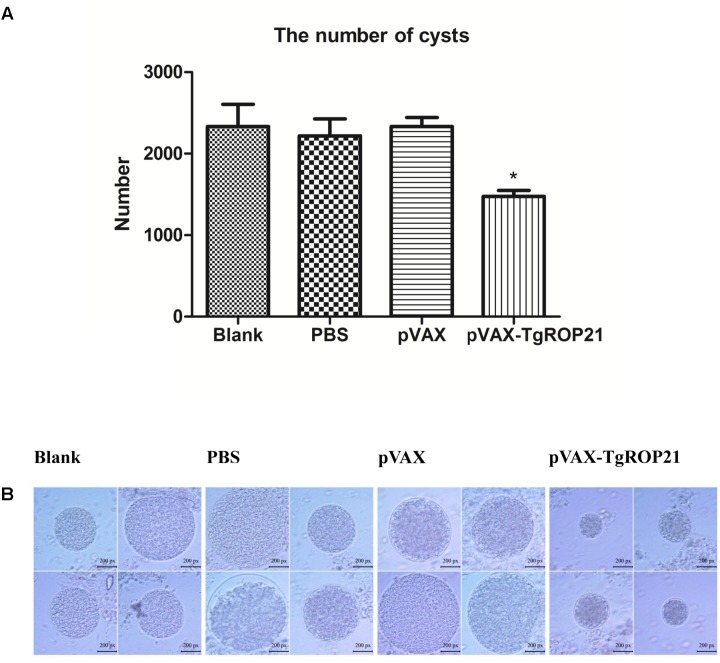
The number and size of brain cysts in the mice after challenge infection with *T. gondii* PRU strain. The number of brain cysts in four groups was determined and the result was expressed as mean ±*SD*. Statistically significant differences (*P* < 0.05), (*P* < 0.01), and (*P* < 0.001) are indicated by (^∗^), (^∗∗^), and (^∗∗∗^), respectively. The size of brain cysts in each group was observed with a microscope. **(A)** The number of brain cysts. **(B)** The size of brain cysts (px: pixel).

In the groups of chronic challenge, one mouth after infection with 10 cysts of *T. gondii* PRU strain, the surviving mice were killed and the cysts in brain were counted. As the result of **Table 1** and **Figure 7A**, the number of brain cysts (1475 ± 163) in the mice immunized with pVAX-TgROP21 decreased significantly (*P* < 0.05) compared to the blank control mice (2333 ± 473). Moreover, the size of brain cysts in the mice immunized with pVAX-TgROP21 was significantly smaller than the groups of blank, PBS, and pVAXI (**Figure 7B**).

**Table 1:** The number and size of brain cysts in the mice after challenge infection with *T. gondii* PRU strain.

Groups	The number of brain cysts (mean ±*SD*)	Cysts decrease ratio (%)	The size of brain cysts (px) (mean ±*SD*)	Size decrease ratio (%)
Blank control	2333 ± 473^a^	0.00^a^	596.60 ± 131.84^a^	0.00^a^
PBS control	2218 ± 413^a^	4.92^a^	583.20 ± 147.39^a^	2.25^a^
pVAX control	2333 ± 191^a^	0.00^a^	595.30 ± 119.32^a^	0.22^a^
pVAX-TgROP21	1475 ± 163^b^	36.78^b^	294.90 ± 76.63^b^	50.57^b^

*In each column, significant difference (P < 0.05) between means and ranks with different letters and no significant difference (P > 0.05) between means and ranks with the same letter.*

## Discussion

Toxoplasmosis caused by *T. gondii* is a severe public problem with a world-wide distribution. In the past studies, the immunogenic efficacy of many *T. gondii* antigens has been extensively confirmed to defend against acute and chronic toxoplasmosis (Kur et al., 2009). As the key role of rhoptry, the ROPs are important in the interaction between the parasite and the host (Bradley et al., 2005). DNA-based vaccination by eukaryotic expression vector, for example, pVAXI, provided an effective approach to protect animals and humans against pathogenic microorganisms, particularly intracellular parasites. The advantages of DNA vaccines are conspicuous since they are easy to be produced and inexpensive with capacity of inducing persistent immune (Gurunathan et al., 2000). So far, lots of work have been done on DNA vaccines against *T. gondii* infection in experiments recently (Chen et al., 2013; Verma and Khanna, 2013; Hassan et al., 2014b). In this work, a recombined DNA vaccine containing ORF of TgROP21 was prepared. It was revealed by the results that this vaccine induced potent immunoreaction and protected the experimental animals against challenge of the highly virulent *T. gondii* RH strain and the chronic *T. gondii* PRU strain.

When *T. gondii* invades host cells, ROPs inside rhoptry organelles are excreted immediately into the infected cell. ROP proteins have an N-terminal signal sequence and a C-terminal hydrophobic sequence that is thought to comprise a transmembrane domain (El Hajj et al., 2006). It was revealed by studies on genetics of *T. gondii* strains with various virulent properties to mice that ROP 15/18 were significant virulent proteins (Behnke et al., 2015; Shwab et al., 2016). ROP48 has eight transmembrane domains and the peptides of ROP48 have good satisfactory flexibility and surface probability. In this study, the protein of TgROP21 was analyzed by DNASTAR, and the result showed that the most regions of TgROP21 protein were hydrophilicity plots and flexible regions and TgROP21 had an excellent antigenic index and surface probability, which indicated the positive antigenicity of TgROP21.

Cytokine exerts a significant role in Th cell activation. IFN-γ which promotes inflammation is not only capable of indicating Th1 cell activation but also related to resisting pathogen invasion (Yano et al., 2002; Cope et al., 2011). It was revealed by results that IFN-γ level was remarkably increased resulting from the stimulation of pVAX-TgROP21, indicating immunoreaction induced by Th1 cells. Th1 cell could produce high levels of IL-2; however, in this study, IL-2 could not be detected. Whether pVAX-TgROP21 could induce Th1-type mediated immunity needed to be further investigated.

Interleukin-4 is known to be a marker cytokine for Th2 cells, and it can promote B cell generation, differentiation, maturation, CD4^+^ cells’ differentiation to Th2 cells, and the production of antibody (Dupont et al., 2012). As one of the cytokines, IL-10 not only exerts multiple roles in inflammatory reaction and immune regulation, but also downregulates co-stimulatory molecule quantity, MHC class II antigen level, as well as Th1 cytokine expression of macrophage cells. IL-10 can enhance B cell survival, proliferation and antibody production, and block NF-κB activity. Moreover, IL-10 is involved in the regulation of the JAK-STAT signaling pathway (Grimbaldeston et al., 2007). In the current study, it was revealed that pVAX-TgROP21 did not induce significantly increased IL-14 or IL-10, indicating that pVAX-TgROP21 could not stimulate Th2-type mediated immunity.

Quan et al. (2012) presented that TgROP1 resulted in significantly increased IL-2, IFN-γ, and TNFα. Sánchez et al. (2011) reported that TgROP2 as vaccine could lead to increased secretion of IFN-γ and IL-10. Mice immunized with pVAX-ROP54 elicited a significant level of Th1-type cytokines (IFN-γ, IL-2, and IL-12p70) and an increased production of Th2-type cytokines (IL-4 and IL-10; Yang et al., 2017). Moreover, as vaccine, TgROP5, TgROP7, TgROP17, TgROP18, and TgROP38 stimulate a Th1-type immune response (Yuan et al., 2011b; Xu et al., 2014a; Wang et al., 2016a,b). In this research, the result of IgG2a over IgG1 demonstrated the capacity of pVAX-TgROP21 vaccination inducing immunoreaction of Th1-type cells. It was also discovered that IFN-γ expression was significantly increased by TgROP21 after initial vaccination and maximized after the third time indicating the capacity of TgROPs stimulating Th1-type immunoreaction.

Specific antibody not only exerts an essential role in cell-mediated immunity but also inhibits the process of parasites adhering to receptors of host cells and kills the parasites inside cells through coating with macrophagocytes (Correa et al., 2007). In the present study, animals immunized with pVAX-TgROP21 exhibited increased anti-*T. gondii* IgG levels in comparison to those of controls (*P* < 0.05). It was uncovered by further experiments on subclasses of IgG that IgG2a was predominant to IgG1, indicating the capacity of pVAX-TgROP21 eliciting humoral immunoreaction mediated by Th1, which was accepted as exerting an essential role in host immune protection against *T. gondii* (Qu et al., 2013; Xu et al., 2014b).

With the aim of evaluating the protective effect of TgROP21 gene vaccine against *T. gondii* infection, vaccinated BALB/c mice received intraperitoneal challenge using 1 × 10^3^ tachyzoites of the highly virulent *T. gondii* RH strain and intragastrically challenged with 10 cysts of the chronic *T. gondii* PRU strain, respectively. Currently, 10^3^ or 10^4^ tachyzoites of *T. gondii* RH strain and 10 or 20 cysts of the *T. gondii* PRU strain were the most widely accepted quantities for developing acute toxoplasmosis model on mice (Chen et al., 2013; Tao et al., 2013; Zhao et al., 2013; Hassan et al., 2014a,c). So we inoculated 10^3^ tachyzoites and 10 cysts in this work.

It was revealed by surviving assays that the surviving duration was remarkably increased due to pVAX-TgROP21 vaccination and pVAX-TgROP22 as vaccine could significantly decrease the number and the size of brain cysts, indicating the capacity of which inducing specific immunoreaction against high virulent and chronic *T. gondii* infection in BALB/c mice model. However, the immunization with pVAX-TgROP21 did not protect the challenged mice from obvious symptoms of toxoplasmosis at later time points and subsequent death, and the approach that TgROP21 as vaccine reduced the number and the size of brain cysts was not clear. Moreover, different strains of Toxoplasma were with different levels of virulence. In our study, we evaluated the immune protection of pVAX-ROP21 by challenging the *T. gondii* RH strain and PRU strain. Whether pVAX-ROP21 can induce protective effect against other strains of *T. gondii* needs to be further researched.

## Conclusion

The eukaryotic expression vector pVAX-TgROP21 was constructed based on the TgROP21 gene published on GenBank by the method of RT-PCR. Immunoblot indicated TgROP21 was expressed in BHK cells. The results of animal challenge experiment revealed that TgROP21 DNA vaccine stimulated potent Th1-type cellular and humoral immunoreaction, extended surviving duration after deadly challenging, and decreased the number and size of cysts. Although TgROP21 merely induced part of protective effect against *T. gondii* acute and chronic infection, it was probably useful for producing vaccines in future researches on anti-*T. gondii* vaccines containing multiple components in mice model. However, whether TgROP21 plays a role in host cell invasion needs to be further studied.

## Author Contributions

ZZ and SW conceived and designed the experiments. YL involved in the preparation of soluble tachyzoite antigens (STAg) and construction of the DNA vaccine plasmid. QX contributed to expression of recombinant plasmids *in vitro*. YL and CW performed BALB/c mice immunization and challenge. SZ, LK, and PL involved in the determination of antibodies by ELISA. MW and SW prepared the figures and tables. ZZ and MW analyzed the data and wrote the paper. All authors read and approved the final manuscript.

## Conflict of Interest Statement

The authors declare that the research was conducted in the absence of any commercial or financial relationships that could be construed as a potential conflict of interest.The reviewer RV and handling Editor declared their shared affiliation.
